# Protein Dynamics Associated with Failed and Rescued Learning in the Ts65Dn Mouse Model of Down Syndrome

**DOI:** 10.1371/journal.pone.0119491

**Published:** 2015-03-20

**Authors:** Md. Mahiuddin Ahmed, A. Ranjitha Dhanasekaran, Aaron Block, Suhong Tong, Alberto C. S. Costa, Melissa Stasko, Katheleen J. Gardiner

**Affiliations:** 1 Linda Crnic Institute for Down Syndrome, Department of Pediatrics, University of Colorado Denver, Mail Stop 8608, 12700 E 19th Avenue, Aurora, Colorado 80045, United States of America; 2 Colorado School of Public Health, University of Colorado Denver, Mail Stop A036-B065 TCH, 12700 E 19th Avenue, Aurora, Colorado 80045, United States of America; 3 Division of Pediatric Neurology, Mail Stop RBC 6090, Department of Pediatrics, Case Western Reserve School of Medicine, Rainbow Babies & Children's Hospital, 11100 Euclid Avenue, Cleveland, OH 44106–6090, United States of America; 4 Department of Biochemistry and Molecular Genetics, University of Colorado Denver, Mail Stop 8608, 12700 E 19th Avenue, Aurora, Colorado 80045, United States of America; 5 Human Medical Genetics and Genomics, and Neuroscience Programs, University of Colorado Denver, Mail Stop 8608, 12700 E 19th Avenue, Aurora, Colorado 80045, United States of America; University of Turin, ITALY

## Abstract

Down syndrome (DS) is caused by an extra copy of human chromosome 21 (Hsa21). Although it is the most common genetic cause of intellectual disability (ID), there are, as yet, no effective pharmacotherapies. The Ts65Dn mouse model of DS is trisomic for orthologs of ∼55% of Hsa21 classical protein coding genes. These mice display many features relevant to those seen in DS, including deficits in learning and memory (L/M) tasks requiring a functional hippocampus. Recently, the N-methyl-D-aspartate (NMDA) receptor antagonist, memantine, was shown to rescue performance of the Ts65Dn in several L/M tasks. These studies, however, have not been accompanied by molecular analyses. In previous work, we described changes in protein expression induced in hippocampus and cortex in control mice after exposure to context fear conditioning (CFC), with and without memantine treatment. Here, we extend this analysis to Ts65Dn mice, measuring levels of 85 proteins/protein modifications, including components of MAP kinase and MTOR pathways, and subunits of NMDA receptors, in cortex and hippocampus of Ts65Dn mice after failed learning in CFC and after learning was rescued by memantine. We show that, compared with wild type littermate controls, (i) of the dynamic responses seen in control mice in normal learning, >40% also occur in Ts65Dn in failed learning or are compensated by baseline abnormalities, and thus are considered necessary but not sufficient for successful learning, and (ii) treatment with memantine does not in general normalize the initial protein levels but instead induces direct and indirect responses in approximately half the proteins measured and results in normalization of the endpoint protein levels. Together, these datasets provide a first view of the complexities associated with pharmacological rescue of learning in the Ts65Dn. Extending such studies to additional drugs and mouse models of DS will aid in identifying pharmacotherapies for effective clinical trials.

## Introduction

Down syndrome (DS) is the most common genetic cause of intellectual disability (ID), affecting approximately one in 750 live births in the United States and one in 1000 live births worldwide [1,2]. While ID can be mild, the average IQ ranges from 40–50 [3,4]. With the improvements in care for people with DS, the average life span, at least in the US, is now 60 years, and the population of people with DS thus continues to increase. With this increase, there is also developing interest in the possibilities for pharmacotherapies to lessen cognitive deficits.

DS is caused by trisomy of all or part of the long arm of human chromosome 21 (Hsa21) and the increased expression, due to dosage, of some subset of the encoded genes. Hsa21 genes that are conserved in mouse include ∼160 encoding diverse protein functions, five microRNAs, and ∼45 encoding keratin associated proteins (KRTAPs) [5]. Hsa21 also encodes several hundred additional genes/gene models of unknown function that lack detectable nucleotide sequence conservation in the mouse genome. A subsegment of Hsa21, labeled the DS Critical Region (DSCR) was proposed to contain genes that were critical to and sufficient for the diagnosis of DS [6]. However, it has been clearly shown that trisomy of other segments, not overlapping with the DSCR, also can result in a diagnosis of DS, including ID [7,8]. Therefore, the DSCR is too limiting a conjecture and genes throughout Hsa21 remain as candidates for contributions to ID.

DS is difficult to model in mice because orthologs of Hsa21 genes map to segments of mouse chromosomes 16, 17 and 10. The most popular and best studied of the many DS mouse models now available is the Ts65Dn [9,10], which is trisomic for the distal segment of Mmu16 spanning 88 orthologs of Hsa21 protein coding genes and 5 microRNA genes [5]. The Ts65Dn is also trisomic for a segment of Mmu17 encoding 50 protein coding genes that are not orthologs of Hsa21 genes [11,12]. While the Ts65Dn therefore is not an ideal model of DS, lacking trisomy of almost 50% of Hsa21 protein coding genes and being trisomic for a substantial set of irrelevant genes, it was the first, and for a long time the only, viable segmental trisomy for an Hsa21 syntenic region. In its more than 20 year history, the Ts65Dn has been shown to display a number of DS relevant neurological phenotypic features [10]. Multiple studies have documented decreased sizes of several brain regions, including the hippocampus and cerebellum, abnormalities in neuron number and dendritic spine morphology, repressed long term potentiation (LTP) and elevated long term depression (LTD), and an age-related loss of functional markers in the basal forebrain cholinergic neurons and adrenergic neurons of the locus coeruleus. Importantly, the Ts65Dn also displays impaired performance in learning and memory (L/M) tasks requiring a functional hippocampus. Such tasks include context fear conditioning (CFC), the Morris water maze (MWM) and novel object recognition (NOR) [10].

Most recently, the Ts65Dn has been used in preclinical evaluations of drugs and small molecules proposed as potential pharmacotherapies for ID in DS. More than 20 drugs have now been shown to rescue, completely or partially, deficits in at least one L/M task, as well as abnormalities in cellular or electrophysiological features [13]. Effective drugs have diverse properties and include γ-aminobutyric acid A (GABAA) receptor antagonists; the N-methyl-D-aspartate receptor (NMDAR) antagonist, memantine; some acetylcholinesterase inhibitors, melatonin, antioxidants; the green tea component, epigallocatechin gallate (EGCG); and the serotonin reuptake inhibitor, fluoxetine [13–21]. The successes of these drug treatments, many of which were carried out in adult mice, have led to considerable enthusiasm for clinical trials. However, for human trials completed to date, outcomes have been modest at best [22–24] and questions remain regarding the validity of preclinical evaluations. For example, while something is known about the target and mechanism of action of each of the drugs tested in the Ts65Dn, information connecting drug action to functions of Hsa21 genes is largely lacking. In addition, preclinical evaluations have not been accompanied by molecular analyses and thus, understanding at the protein level of how these drugs work in the Ts65Dn, and how this may or may not be reflected in rescue of ID in DS due to full trisomy Hsa21, is completely unknown.

Here, we seek to address some of these issues by examining the protein responses in the Ts65Dn that have been treated with memantine. Memantine is an uncompetitive antagonist of the NMDAR and binds the NR2A and NR2B subunits with low affinity and high on and off rates [25,26]. Its unique properties allow it to modulate NMDAR mediated excitatory neurotransmission without the negative side effects seen with other NMDAR antagonists. Memantine has been approved for use in moderate to severe Alzheimer’s Disease (AD) [27] and interest in its possible use in DS originally stemmed from functional information on one Hsa21 encoded protein, regulator of calcineurin 1, RCAN1. RCAN1 was shown to modulate the activity of the Ca^++^-calmodulin protein phosphatase, calcineurin (CaN) [28]. Because dephosphorylation by CaN decreases the probability of NMDAR opening [29], a simple hypothesis for DS was that RCAN1 overexpression inhibits CaN activity, leading to hyperphosphorylation and hyperactivation of NMDAR, which in turn leads to neurotoxicity. This hypothesis was supported by observations of hyper-responsiveness of the Ts65Dn to the locomotor stimulatory effects to the NMDAR antagonist, MK-801 [16]. The hypothesis is, however, an oversimplification of the consequences of trisomy because several Hsa21-encoded proteins have been shown individually to directly or indirectly impact NMDAR activity [30]. Among these, the activity of RCAN1 is modulated by phosphorylation carried out in part by the Hsa21 kinase DYRK1A [31], and another Hsa21 protein, PCP4 (Purkinje cell protein 4), modulates activity of calmodulin [32], with consequences for the dynamics of CaN activity. Additional Hsa21 proteins interact with the NMDAR: the multidomain protein Intersectin1 (ITSN1) that is involved in endocytosis, the guanine nucleotide exchange factor TIAM1, and the amyloid precursor protein, APP, that is mutated or duplicated in some familial AD [33–35]. It is not possible to predict the consequences for the dynamics of NMDAR activity caused by the concurrent overexpression of all of these proteins, however, Costa et al [16] pursued the hypothesis of hyperactive NMDAR contributing to L/M deficits in the Ts65Dn and demonstrated that acute injection of memantine rescued impaired performance of the Ts65Dn in CFC. Subsequently, memantine was also shown to rescue impairments in the Ts65Dn in the acquisition phase of the MWM and in NOR, to partially rescue performance in the water radial arm maze, and to rescue abnormal levels of LTD [36–38]. In spite of these successes, however, clinical trials were unsuccessful [22,23].

Here, we describe the effects of memantine on protein expression in brain regions of Ts65Dn mice exposed to CFC. We have previously described in control mice the responses in 85 proteins/protein modifications to both normal learning in CFC and to memantine [39]. Here we show that Ts65Dn that have not been trained in CFC already have elevated levels of approximately half the proteins that respond when control mice successfully learn. Furthermore, the dynamic responses seen in control mice largely do not occur in untreated Ts65Dn, at least within the time frame assayed, consistent with their failure to learn. Treatment with memantine does not normalize the initial levels but does result in normalization of the endpoint levels after successful learning. We describe patterns in protein responses to normal, failed and rescued learning, and to memantine, and begin to identify critical changes and abnormalities that must be corrected in DS for successful learning to occur.

## Materials and Methods

### Ethics Statement

All procedures were approved by the Institutional Animal Care and Use Committee of the University of Colorado (B-58111(01)1E) and were performed in accordance with National Institutes of Health guidelines for the care and use of animals in research.

### Experimental animals

Ts65Dn mice are on a mixed C57BL/6JEi x C3Sn.BLiA (B6EiC3Sn.BLiA F2) background (statistically 50% B6Ei; 50% C3) obtained by breeding B6EiC3Sn.BLiA *a*/A-Ts65Dn females and B6EiC3Sn.BLiA F1 males [9,40]. Mice were bred at the University of Colorado School of Medicine (Aurora, Colorado) or The Jackson Laboratory (Bar Harbor, Maine). Colonies were maintained in a room with HEPA-filtered air and a 14:10 light:dark cycle, fed a 6% fat diet and acidified (pH 2.5–3.0) water *ad libitum*. Littermates (S1 Table) were housed in the same cage. Ts65Dn mice were genotyped by quantitative (real time) polymerase chain reaction (qPCR) for genes in the trisomic segment [41]. Male mice only (age 3–4 months) were used. The wild type littermates have been reported previously [39].

### Context fear conditioning

Context fear conditioning (CFC) was performed as described [16,39,42]. Briefly, mice were placed in a novel cage (Med Associates, St. Albans, VT, Modular Mouse Test Chamber), allowed to explore for three minutes and then given an electric shock (2 s, 0.7 mA, constant electric current). These mice are the context-shock (CS) group and learn to associate the context with the aversive stimulus [42]. Learning is displayed by “freezing” upon re-exposure to the context, where freezing is defined as a lack of movement except for respiration. A second group of mice were placed in the novel cage, immediately given the electric shock (2 s), and then allowed to explore for 3 min. These mice are the shock-context (SC) group and do not acquire conditioned fear. All mice received an injection of memantine (5mg/kg i.p.) or the equivalent volume of saline 15 minutes prior to exposure to the novel context. Eight groups of mice (7–10 per group) were used: trisomic and controls, CS mice injected with saline or with memantine and SC mice injected with saline or with memantine, as described [16]. Mice were sacrificed at 60 minutes post training without measurement of freezing; prior work has demonstrated that CS control mice of this age and strain background, and with this CFC protocol, are successful in learning, CS Ts65Dn mice injected with saline and all SC mice do not learn, CS Ts65Dn mice injected with memantine learn as well as control mice, and memantine does not affect freezing [16].

### Tissue processing and preparation of protein lysates

Mice were sacrificed by cervical dislocation, without anesthetic to minimize additional alterations to the protein profiles. Brain dissections for hippocampus and cortex, and preparation of protein lysates from subcellular fractions (nuclear-enriched, cytosolic and crude membrane) have been described [39]. Protein concentrations, determined using the 660 nM Protein Assay kit (Pierce), were within the range of 3–4 mg/ml for all samples. Information for each mouse, including for the control mice reported previously [39], on age, littermates, and weights of individual brain regions is provided in S1 Table.

### Antibodies and validation for RPPA

Antibodies are listed in S2 Table. Prior to use in RPPA, each lot of each antibody was tested on Western blots of mouse brain lysates, as described previously [43], and verified to produce predominant band(s) of explainable size, i.e. clean band(s) of the correct size(s) in the absence of significant background and non-specific bands. All antibodies listed in S2 Table are suitable for RPPA.

### Array assembly and printing

For each sample, a five point dilution series in three replicates was printed as described previously [43]. Arrays with nuclear-enriched and cytosolic fractions from hippocampus from all mice (controls and trisomy together) were produced in three print runs and those from cortex, in two print runs. Membrane fractions from cortex and hippocampus were printed on the same slides in two print runs. Slides were stored at 4°C until use.

### Slide screening and data analysis

Methods for screening and processing of slides with antibodies, normalization of signal intensities to those obtained with the general protein stain SyproRuby (Invitrogen, CA, USA), and analysis of protein expression data have been described [39]. Data from antibody screenings that produced normalized signal intensities <0.1 were considered too low to be reliable and were discarded. Additional details of quality control and validation of inter-slide and inter-print run reproducibility have been described [43].

### Statistical analysis

After exclusion of technical outliers, each SyproRuby-normalized protein value was included in the statistical analyses if the level was within 3 standard deviations of the mean. For each protein, mean differences were calculated for the biologically relevant, pairwise group comparisons listed in Table 1. Treatment and genotype differences were assessed using a three-level mixed effects model to account for possible correlations among replicates and dilution levels within each mouse, with different mice being the random effects. To control for multiple testing, Bonferroni corrections were applied. S3 Table contains results (% difference, p-value and SEM) for all proteins in the nuclear, cytosolic and membrane fractions, for each of the pairwise comparisons. Analyses were carried out using SAS version 9.3 (SAS Institute Inc., Cary, NC).

**Table 1:** Biologically relevant pairwise group comparisons.

Comparison		Groups	Biological interpretation
Initial condition, baseline	B	t-SC-sal vs. c-SC-sal	Initial genotype differences
Initial conditions, trisomy	B-tm	t-SC-mem vs. t-SC-sal	Effects of memantine on trisomy baseline
Initial conditions control	B-cm	c-SC-mem vs. c-SC-sal	Effects of memantine on control baseline
Initial conditions	B-tm-cs	t- SC-mem vs. c-SC-sal	Effects of memantine on genotype differences
Normal learning	NL	c-CS-sal vs. c-SC-sal	Effects of CFC training in saline-injected controls
Failed learning	FL	t-CS-sal vs. t-SC-sal	Effects of CFC training in saline-injected Ts65Dn
Rescued learning	RL	t-CS-mem vs t-SC-mem	Effects of CFC training in memantine-injected Ts65Dn
Endpoint rescued vs failed	RL-FL	t-CS-mem vs. t-CS-sal	Differences in final profiles
Endpoint rescued vs. normal	RL-NL	t-CS-mem vs. c-CS-sal	Differences in final profiles

B, baseline; SC, shock-context; CS, context-shock; t, trisomic (Ts65Dn); c, control; tm, trisomy injected with memantine; cm, control injected with memantine; cs, control injected with saline; sal, saline; mem, memantine

## Results

The goals of these experiments were to determine the changes in protein profiles in the Ts65Dn mice after failed learning in CFC, how these changes differ from the normal responses in control mice that exhibit successful learning, and how these changes are modified in Ts65Dn when learning is rescued by memantine. The data reported here were generated in parallel with those previously reported in control mice subjected to CFC with and without memantine [39], and used littermates of those mice. Levels of 85 proteins/protein modifications (listed in S2 Table) were assayed in nuclear, cytosolic and/or membrane fractions from hippocampus and cortex. Seven proteins were encoded by Hsa21. Those not encoded by Hsa21 included 20 and 14 components of the MAPK and MTOR pathways, respectively, 14 subunits of glutamate receptors and their interaction partners, 7 proteins involved in apoptosis or inflammation, four immediate early gene (IEG) proteins, three histone modifications, and 12 related to abnormalities seen in brains of Alzheimer’s Disease (AD) and/or mouse models of AD. Twenty proteins were site-specifically phosphorylated forms. Proteins were chosen because abnormalities have been observed in DS or DS models, and/or because they are known or predicted to be relevant to pathways involved in LM or synaptic plasticity.

**Fig 1:**
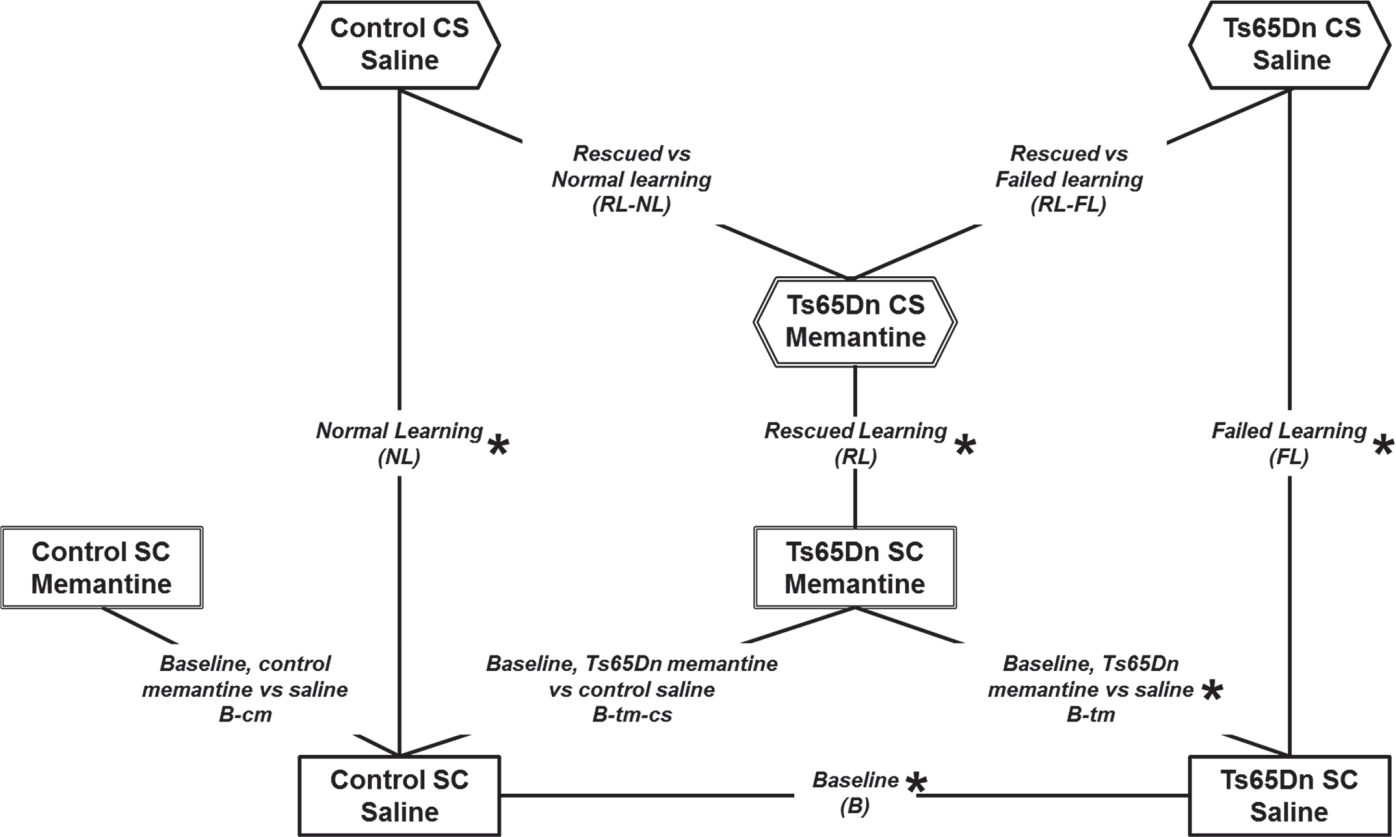
Relationships among pairwise group comparisons. Eight groups of mice, control and Ts65Dn, not trained in CFC (SC, shock context) and trained in CFC (CS, context shock), injected with either saline or memantine, were generated. The four SC groups represent baseline (B) conditions and are shown in rectangles in the lower part of the figure. Three CS groups are shown in hexagons. Lines connecting groups indicate the nine pairwise comparisons of biological interest that are discussed here. Each comparison is labelled with the complete name and the abbreviated name used in the text. Summary data for all comparisons, except B-cm, are presented in Table 2 (for B-cm data, see reference 39). *, comparisons analyzed for data shown in Figs. 2–7. See Table 1 for additional information.

A standard protocol for CFC requires two groups of mice, the SC (shock-context) group that does not learn the context and the CS (context-shock) group that should learn. For the experiments here, one half of each of the SC and CS groups of mice were injected with saline and the other half, with memantine. Thus, for the two genotypes, control and trisomic, there are a total of 8 groups of mice. Differences due to genotype and changes caused by treatment were determined by pairwise group comparisons. Twenty-eight pairwise comparisons can be done but only a subset of these are of biological interest. Fig. 1 illustrates the relationships between groups that form the subset of comparisons discussed here. The nomenclature is complicated and listed for reference in Table 1. Four comparisons concern initial conditions: B (baseline; Ts65Dn-saline vs. control-saline, t-SC-s vs. c-SC-s) identifies initial abnormalities in the Ts65Dn relative to controls when neither is trained in CFC; B-tm (baseline Ts65Dn treated with memantine) and B-cm (baseline control treated with memantine) identify changes in the initial protein profiles (SC-m vs. SC-s) in Ts65Dn and control mice, respectively, caused by memantine treatment alone, and B-tm-cs (Ts65Dn treated with memantine vs. controls treated with saline, t-SC-m vs. c-SC-s) shows how well or poorly memantine treatment normalizes the baseline, initial, Ts65Dn profile. Three comparisons represent changes associated with stimulation to learn: NL (normal learning in control mice; c-CS-s vs. c-SC-s), FL (failed learning in Ts65Dn mice; t-CS-s vs. t-SC-s), and RL (rescued learning in Ts65Dn mice; t-CS-m vs. t-SC-m). Lastly, two comparisons represent “end points”: RL-FL (t-CS-m vs. t-CS-s) identifies differences in the final profiles between rescued and failed learning in the Ts65Dn mice and RL-NL (t-CS-m vs. c-CS-s) identifies how well, or poorly, profiles in rescued learning in the Ts65Dn resemble those in normal learning in control mice. Two of these comparisons, NL and B-cm, were reported previously and are included here for ease of comparison.

Of 85 proteins screened in one or more fractions from hippocampus, levels of expression of 73 and 66 were detectable in nuclear and cytosolic fractions, and 28 in membrane fractions. For technical reasons, fewer proteins were detected in nuclear and cytosolic fractions from cortex, 66 and 55, respectively. General features of protein responses, the number and direction of changes, are summarized in Table 2. In hippocampus, in all three subcellular fractions, in B, levels of 30%-50% of proteins are abnormal and the large majority are elevated with respect to controls. The nuclear fraction shows the most abnormalities, with levels of 41 proteins elevated (and none repressed). Notably, memantine treatment did not rescue the abnormalities, rather it increased to 50 the number of abnormalities in the Ts65Dn when compared to saline treated controls (B-tm-cs). In cortex, it is the cytosolic fraction that shows the most changes, with 42 abnormalities in B and 31 abnormalities after memantine treatment (B-tm-cs), while in the nuclear fraction, only 25 and 19 proteins were affected by trisomy and by memantine treatment, respectively. Cortex also showed overall a larger proportion of repressed protein levels.

**Table 2:** Summary of protein responses to CFC and memantine.

	NL	FL	B	RL	B-tm	B-tm-cs	RL vs FL	RL vs NL
Hippocampus								
Nuclear	36+, 3−	6+, 20−	41+, 0−	6+, 20−	17+, 2−	50+, 0−	22+, 0−	5+, 2−
Cytosolic	23+,14−	21+,11−	20+, 3−	23+, 2−	7+, 10−	21+,5−	3+, 5−	11+, 4−
Membrane	8+, 6−	3+, 9−	6+, 2−	6+, 7−	4+, 5−	9+, 5−	7+, 0−	3+, 0−
Cortex								
Nuclear	14+, 13−	7+,20−	20+, 5−	9+, 38−	14+, 8−	19+, 0−	2+,10−	2+, 13−
Cytosolic	25+, 13−	6+, 10−	30+, 12−	19+, 19−	15+, 12−	28+, 3−	7+, 4−	10+,4−
Membrane	3+, 12−	4+, 7−	6+, 12−	2+, 20−	19+, 4−	16+, 0−	3+, 6−	3+, 1−

Number of proteins that increased (+) and decreased (−) in response to treatment or that differed in Ts65Dn from controls with treatment. Abbreviations as in Table 1.

In FL, there were fewer responses than in NL and a greater proportion were decreases. For example, in the nuclear fraction of hippocampus, in FL, there were only six increases and 20 decreases, while in NL there were 36 increases and three decreases. In the cytosolic fraction of cortex in FL, there were only six increases and 10 decreases, while in NL, there were 25 increases and 13 decreases. In RL, the number of responses more closely resembles FL (e.g. only three increases and 17 decreases in the hippocampal nuclear fraction) and does not suggest correction of the responses to those seen in NL. Nevertheless, in comparing the endpoints of successful learning in memantine-treated Ts65Dn with normal learning saline-treated controls (RL vs NL), the initial genotype differences of 41 elevated proteins were reduced to only five elevated and two repressed in the nuclear fraction of hippocampus.

Overall, these data show that protein profiles in the Ts65Dn are abnormal in initial, baseline, conditions and that dynamic responses are fewer than, and often opposite to, those in normal learning at this time point (60 minutes) post training. Memantine treatment does not correct the initial abnormalities and does not induce the dynamic responses seen in NL, but does result in normalization of endpoint profiles, after rescued learning. In the following sections, we first discuss patterns in protein responses in NL, FL, B, RL and B-tm (Table 3) and use them to predict which abnormalities in B and responses in FL contribute to impaired learning. We then describe some of the specific proteins, pathways and complexes that are most significantly affected by the perturbations in trisomy and rescue with memantine. Complete data sets are available in S3 Table.

**Table 3:** Protein responses and differences in hippocampus.

Nuclear fraction							
	NL	FL	B	RL	B-tm	B-tm-cs	RL v NL
Protein	Normal	Failed	Baseline	Rescued	Ts SC mem vs Ts SC sal	Ts SC mem vs C SC sal	Ts CS mem vs C CS Sal
AKT	5.8%	−12.8%	15.5%	−11.1%	−2.7%	12.4%	−5.5%
AMPKA	11.1%	−2.7%	5.5%	4.1%	0.3%	5.9%	−0.9%
APP	9.0%	−8.2%	**36.4%**	4.1%	−3.2%	**32.0%**	**26.0%**
ARC	3.5%	**−6.1%**	3.6%	−4.1%	6.3%	**9.1%**	1.9%
BAD	−0.9%	**−12.3%**	3.6%	−1.6%	6.2%	**10.2%**	9.4%
BAX	**8.0%**	−5.8%	7.4%	−1.5%	−2.6%	4.6%	−4.6%
BCL2	−2.2%	**−18.6%**	9.4%	−4.2%	−5.8%	3.0%	1.0%
BDNF	3.5%	−5.9%	1.3%	−7.5%	**10.9%**	**12.3%**	0.3%
BRAF	**59.4%**	**26.7%**	**29.0%**	**40.6%**	−9.9%	16.2%	2.6%
CAMKII	**12.3%**	**0.2%**	**12.7%**	**−12.4%**	6.7%	**20.2%**	−6.2%
CaNA	**16.7%**	**19.7%**	2.3%	**22.8%**	2.4%	4.7%	**10.2%**
CASP3	6.9%	**−19.8%**	**20.1%**	−8.4%	−1.0%	**19.0%**	2.0%
CDK5	5.1%	4.7%	−3.6%	1.8%	8.9%	5.0%	1.6%
CFOS	5.5%	**−9.4%**	6.7%	−4.4%	4.1%	**11.0%**	0.6%
CREB	−1.8%	−6.9%	−1.8%	**−16.9%**	**19.6%**	**17.4%**	−0.7%
CTNNB1	**11.3%**	−1.4%	1.2%	−2.6%	9.8%	11.2%	−1.5%
DYRK1A	6.3%	3.0%	**21.0%**	−0.5%	3.8%	**25.7%**	17.6%
EGR1	6.8%	−6.0%	7.8%	**−12.2%**	9.4%	**18.0%**	−3.0%
ELK	**34.0%**	−1.1%	**21.3%**	10.1%	−11.0%	8.0%	**−11.3%**
ERBB4	**8.5%**	−4.1%	**8.0%**	**−7.9%**	**12.3%**	**21.3%**	2.9%
ERK	**21.1%**	5.5%	**16.9%**	**70.5%**	−35.5%	−24.6%	6.1%
FYN	**20.2%**	**11.0%**	**14.1%**	−7.8%	**12.6%**	**28.5%**	−1.4%
GAD2	−1.2%	**−16.7%**	5.1%	**−12.1%**	**13.0%**	**19.0%**	5.9%
GFAP	0.4%	**−20.8%**	10.0%	**−18.2%**	**10.6%**	**21.7%**	−0.8%
GSK3B	**25.5%**	**14.8%**	**13.5%**	29.2%	−16.1%	−4.8%	−2.0%
H3AcK18	**−30.9%**	**−44.9%**	**25.4%**	**−43.8%**	−7.7%	15.7%	−5.9%
H3AcK9	**−42.1%**	**−43.6%**	15.4%	**−44.5%**	−11.4%	2.3%	−1.9%
H3MeK4	−9.4%	**−28.6%**	**18.3%**	**−19.9%**	−6.5%	10.7%	−2.1%
IL1B	4.4%	−4.8%	5.9%	−2.1%	4.6%	**11.1%**	4.2%
ITSN1	8.3%	−0.3%	**30.4%**	−0.4%	2.0%	**33.0%**	**22.4%**
JNK	**19.2%**	−5.1%	**17.4%**	−2.8%	−1.9%	**15.2%**	−5.7%
MEK	7.6%	−4.0%	**11.3%**	0.3%	−9.9%	0.3%	−6.5%
MTOR	2.7%	−6.9%	**7.9%**	**−11.6%**	6.8%	**15.3%**	−1.2%
nNOS	6.2%	−8.8%	6.0%	1.7%	8.8%	**15.3%**	10.4%
NUMB	**16.1%**	3.6%	5.1%	−4.3%	**20.3%**	**26.4%**	4.2%
P3525	**15.3%**	−2.1%	8.0%	5.1%	−3.4%	4.3%	−4.9%
P70S6	**21.0%**	−3.7%	**14.9%**	−0.8%	9.8%	**26.2%**	3.5%
pAKT	**13.2%**	−6.6%	**13.2%**	−2.5%	5.7%	**19.6%**	3.1%
pBRAF	10.8%	**−12.9%**	**14.8%**	−4.9%	3.5%	**18.7%**	2.0%
pCAMKII	**120.6%**	8.4%	**41.7%**	−5.7%	**48.2%**	**110.0%**	−10.2%
pCASP9	**19.6%**	2.7%	**19.6%**	−8.3%	**13.2%**	**35.4%**	3.8%
pCFOS	**13.0%**	3.4%	**7.7%**	−4.9%	8.2%	**16.6%**	−1.9%
pCREB	5.0%	**−12.8%**	6.2%	**−13.8%**	**15.4%**	**22.5%**	0.7%
pEIF4B	**11.9%**	**−13.9%**	**19.4%**	−4.3%	−1.2%	**18.0%**	1.0%
pELK	−8.2%	−9.1%	0.5%	0.8%	7.2%	7.7%	18.2%
pERK	**58.6%**	**13.8%**	**28.9%**	**22.4%**	4.6%	**34.8%**	4.0%
pGSK3BS9	**13.1%**	−10.4%	**12.7%**	−0.6%	−2.9%	**9.5%**	−3.7%
pGSK3BT216	7.6%	2.3%	3.5%	−4.5%	**12.1%**	**16.1%**	3.0%
pJNK	**36.3%**	7.9%	10.6%	**−11.6%**	**42.6%**	**57.7%**	2.2%
PKCA	**26.6%**	4.1%	**20.4%**	−1.4%	1.9%	**22.7%**	−4.5%
pMEK	**20.8%**	−1.0%	**17.5%**	0.5%	−5.7%	**10.7%**	−8.2%
pMTOR	**20.1%**	−0.5%	**14.4%**	−6.0%	8.9%	**24.5%**	−2.6%
pNUMB	**31.7%**	0.0%	**19.9%**	**−8.9%**	8.5%	**30.1%**	**−9.9%**
PP2A	**10.2%**	−2.0%	**7.4%**	**21.0%**	**−14.4%**	−8.0%	1.0%
pP70S6	−7.0%	−20.9%	12.9%	**−24.2%**	5.0%	18.6%	−3.3%
pPKCA	**33.3%**	−5.7%	**30.8%**	0.3%	2.1%	**33.5%**	0.5%
pPKCG	3.4%	1.6%	4.1%	7.8%	9.6%	14.1%	19.0%
pRSK	**23.1%**	−6.6%	**18.5%**	−1.0%	8.6%	**28.6%**	3.4%
pS6	**20.9%**	0.8%	**22.8%**	−1.0%	**14.8%**	**40.9%**	**15.4%**
pSRC	**36.7%**	**31.1%**	3.1%	9.0%	**27.0%**	**30.9%**	4.4%
RAPTOR	6.6%	**−12.3%**	7.5%	−1.1%	2.2%	**9.8%**	1.8%
RCAN1	4.2%	−5.4%	**21.6%**	5.0%	−5.3%	**15.1%**	19.3%
RRP1	−9.8%	−11.6%	16.9%	6.9%	−12.4%	2.4%	21.3%
RSK	**16.9%**	−10.4%	**14.6%**	10.8%	−7.8%	5.6%	0.1%
S6	14.8%	5.0%	**14.0%**	8.9%	7.9%	**23.0%**	16.7%
SHH	−3.1%	**−16.2%**	2.6%	**−20.2%**	**15.4%**	**18.5%**	−2.4%
SNCA	−0.5%	**−14.1%**	3.2%	**−14.8%**	**12.0%**	**15.6%**	−1.0%
SOD1	**−45.4%**	**−60.6%**	**32.9%**	**−54.6%**	**−10.1%**	**19.5%**	0.0%
TAU	−11.9%	**−17.8%**	16.6%	**−27.4%**	12.0%	30.6%	7.6%
TH	**12.8%**	**−13.2%**	**14.8%**	1.9%	−3.0%	**10.9%**	0.6%
TIAM1	−6.4%	−7.5%	6.0%	1.1%	1.6%	**7.7%**	**16.3%**
TRKA	**15.2%**	−4.7%	**14.9%**	**21.6%**	−16.1%	−3.6%	1.8%
Ubiquitin	**13.5%**	−5.1%	**12.3%**	**−5.9%**	**9.2%**	**22.7%**	1.8%
Cytosolic fraction							
AKT	**16.8%**	2.0%	**8.4%**	5.4%	−3.2%	5.0%	−5.2%
AMPKA	4.0%	2.7%	7.2%	**13.5%**	−6.6%	0.1%	9.2%
ARC	0.0%	7.6%	−3.5%	9.5%	1.7%	−1.9%	7.4%
BAD	**−20.7%**	**−19.6%**	11.6%	3.2%	**−22.7%**	−13.7%	12.3%
BAX	0.9%	3.5%	2.2%	−2.2%	−0.4%	1.7%	−1.3%
BCL2	**−15.4%**	−4.8%	−0.3%	4.9%	−6.2%	−6.6%	**15.7%**
BDNF	**12.0%**	**28.7%**	−3.1%	3.9%	6.3%	3.0%	−4.4%
BRAF	12.8%	5.4%	**26.8%**	20.8%	−15.9%	6.7%	14.3%
CAMKII	6.3%	5.2%	4.0%	**7.2%**	−5.6%	−1.9%	−1.0%
CaNA	**−28.1%**	−5.7%	−8.4%	−0.7%	−4.2%	**−12.2%**	**21.2%**
CASP3	**−13.8%**	−1.8%	5.4%	3.7%	−2.2%	3.1%	**24.0%**
CDK5	**9.5%**	**18.9%**	−3.7%	**14.4%**	1.1%	−2.6%	1.6%
CTNNB1	**−18.3%**	−7.8%	−8.2%	**17.7%**	**−16.5%**	**−23.4%**	10.3%
DYRK1A	18.4%	11.2%	**31.8%**	**24.1%**	−11.7%	16.4%	**22.0%**
EGR1	**19.5%**	**12.0%**	8.7%	**17.7%**	−9.1%	−1.3%	−3.0%
ELK	**30.3%**	0.3%	9.2%	**11.0%**	0.1%	**9.2%**	−6.9%
ERBB4	4.4%	−4.3%	3.6%	1.3%	−3.3%	0.1%	−2.8%
ERK	**17.3%**	2.6%	**13.7%**	**11.2%**	−5.0%	8.0%	2.4%
FYN	**−29.6%**	−9.1%	−6.9%	−11.7%	−4.0%	−10.6%	12.0%
GAD2	**−20.9%**	−12.2%	−2.8%	1.0%	−7.4%	−10.0%	14.8%
GluR4	7.9%	1.5%	10.3%	11.9%	**−12.4%**	−3.7%	−1.6%
GSK3B	**32.1%**	**11.2%**	**13.7%**	**12.1%**	3.2%	**17.4%**	−0.3%
IL1B	**−17.9%**	15.2%	**−18.1%**	**−15.9%**	**28.6%**	5.2%	7.7%
ITSN1	6.5%	−3.1%	**31.7%**	11.8%	−9.8%	**18.7%**	**24.6%**
JNK	**30.8%**	**16.3%**	**8.1%**	**35.5%**	**−16.0%**	−9.2%	−6.0%
MEK	**25.2%**	**22.0%**	−1.6%	4.7%	5.9%	4.2%	**−12.8%**
MTOR	**26.4%**	**20.8%**	1.9%	**9.2%**	**8.8%**	**10.9%**	−4.2%
nNOS	−17.1%	1.5%	0.1%	3.5%	1.4%	**1.4%**	26.7%
NR2A	4.8%	**31.6%**	−12.9%	**85.8%**	**−26.4%**	**−35.8%**	**13.8%**
NR2B	**34.4%**	**14.7%**	**15.1%**	**14.3%**	−2.4%	**12.4%**	−4.4%
NUMB	10.6%	9.0%	−1.0%	5.9%	2.1%	0.7%	−3.6%
P3525	**−18.6%**	**−16.4%**	−8.2%	−1.7%	−9.7%	**−17.0%**	0.1%
P70S6	**13.4%**	−5.4%	**22.6%**	−7.0%	0.8%	**23.6%**	1.2%
pAKT	4.0%	−2.1%	8.4%	−5.6%	7.5%	**16.5%**	5.7%
pBRAF	**25.2%**	**13.9%**	**11.8%**	**9.0%**	5.6%	**18.1%**	2.8%
pCAMKII	**125.3%**	36.5%	13.7%	46.6%	42.6%	**62.1%**	5.4%
pCASP9	4.7%	4.3%	12.8%	−16.1%	18.9%	**34.1%**	7.4%
pEIF4B	**−22.1%**	**−23.6%**	4.5%	0.5%	−18.2%	−14.5%	10.5%
pELK	−6.1%	−7.1%	15.4%	−2.2%	−5.6%	8.9%	13.4%
pERK	**194.4%**	**154.7%**	9.5%	**108.0%**	7.6%	17.8%	−16.8%
pGSK3BT216	6.2%	**12.5%**	5.6%	3.6%	**8.2%**	**14.2%**	11.4%
pJNK	**19.9%**	**27.1%**	**11.4%**	**11.3%**	11.1%	**23.7%**	14.8%
PKCA	−16.0%	4.0%	−8.9%	−15.8%	−5.6%	−14.0%	−13.8%
pMEK	**47.8%**	**35.8%**	**−11.8%**	**21.8%**	12.7%	−0.7%	**−18.2%**
pMTOR	**32.7%**	**20.3%**	**9.2%**	**14.1%**	6.1%	**15.8%**	−0.5%
pNR1	**23.7%**	**16.1%**	4.6%	**−0.7%**	**14.6%**	**19.9%**	−3.8%
pNR2A	**40.3%**	**25.3%**	1.3%	**12.9%**	**13.4%**	**14.8%**	**−7.7%**
pNR2B	**14.1%**	8.6%	0.9%	**25.1%**	−2.0%	−1.2%	**8.4%**
pNUMB	**154.6%**	40.3%	8.1%	**44.8%**	**30.2%**	**40.8%**	**−20.0%**
PP2A	**−14.5%**	0.0%	−7.1%	−2.4%	−1.0%	−8.0%	5.0%
pP70S6	**−25.1%**	**52.8%**	**−28.5%**	**−25.8%**	**32.5%**	−5.2%	−6.2%
pPKCA	11.9%	**−31.7%**	**36.1%**	−10.3%	−9.1%	**23.7%**	−0.8%
pRSK	−1.7%	**−16.7%**	**19.7%**	8.9%	**−17.2%**	−0.9%	9.8%
pS6	−8.4%	**−27.5%**	17.7%	7.7%	**−19.9%**	−5.6%	11.0%
PSD95	−4.4%	10.5%	0.6%	4.6%	−1.4%	−0.7%	8.6%
RAPTOR	**15.9%**	**18.1%**	3.7%	**13.2%**	−5.6%	−2.3%	−4.7%
RCAN1	0.4%	**−9.0%**	**26.7%**	7.3%	−7.9%	**16.6%**	**24.4%**
RRP1	3.3%	**−14.5%**	**13.9%**	12.1%	**−15.8%**	−4.1%	4.0%
RSK	4.4%	−3.2%	7.8%	12.0%	−14.6%	−8.0%	−0.5%
S6	**−25.4%**	**−36.3%**	**6.6%**	**36.7%**	**−38.3%**	**−34.2%**	20.5%
SOD1	−5.7%	**−18.9%**	**58.1%**	6.0%	**−25.3%**	**18.1%**	**32.8%**
TAU	8.8%	−4.3%	**31.3%**	5.0%	−8.1%	**20.7%**	16.4%
TH	−1.6%	−7.4%	7.1%	2.1%	−8.9%	−2.4%	1.2%
TIAM1	6.4%	−1.0%	**33.5%**	−0.4%	4.0%	**38.9%**	**30.1%**
TRKA	−2.9%	2.0%	**−6.7%**	6.8%	0.7%	−6.1%	3.2%
Ubiquitin	9.3%	**11.8%**	0.3%	1.6%	6.6%	6.8%	−1.1%

Significant changes/differences, after Bonferroni correction (p< 0.0007 to p<10e-22), are indicated in **Bold**. For additional comparisons and complete datasets, see S3 Table.

### Patterns in protein responses

To gain insight into which abnormalities in the Ts65Dn in B and FL are relevant to impaired learning and therefore must be corrected by memantine (or other drug treatment) to facilitate L/M, we examined the pattern of protein responses from five comparisons, NL, FL, RL, B and B-tm. The large number of measurements allows the grouping of protein responses into potentially informative patterns. For this analysis, we define an instance as the set of measurements in NL, FL, B, RL and B-tm for one protein in one fraction from a brain region. There are therefore 73, 66 and 28 instances from detection of proteins, respectively, in the nuclear, cytosolic and membrane fractions from hippocampus and 66, 55 and 29, from cortex.

To classify patterns, we first consider events occurring in NL and assume that each is required for successful learning. When a protein increases or decreases in NL, then either the actual dynamic response is required, i.e. a change in level is necessary, or a specific level of the protein is required and in control mice this is achieved by a change. Patterns of associated responses seen in FL, RL and B, when there are changes in NL, can then be interpreted as follows: (i) if the same changes occur in FL and in RL, then, while these responses may be necessary, they are not sufficient for successful learning; (ii) if the same changes do not occur in FL or in RL, but the protein level is abnormal in B and already similar to the level produced by NL, then specific *levels* of these proteins are adequate for successful learning and the levels in B can compensate for the changes occurring in NL; and (iii) if the changes that occur in FL and RL are smaller than those in NL but levels in B are also abnormal, then the latter are partially compensating, such that B + FL and B + RL are equivalent to the changes in NL

Responses in the foregoing patterns are adequate in the Ts65Dn without memantine treatment and therefore are not contributing to learning impairment. However, the untreated Ts65Dn fail to learn, and therefore some of the responses seen in NL do not occur in FL and are not compensated. These include instances where NL ≠ 0 and FL = 0, with the following patterns: (iv) no changes are seen in B-tm, but changes occur in RL such that RL ≈ NL; therefore, memantine indirectly induces the required responses; (v) changes are absent in RL, but memantine directly induces the required changes, i.e. B-tm = NL; again, similar to (ii) above, it is the levels of the proteins that are adequate for successful learning and no further changes in RL are required; and (vi) changes occur in RL that are not equivalent to those in NL and levels in B and/or B-tm partially compensate; in these cases, B + B-tm + RL = NL. In each of these three patterns, the lack of response or inadequate response in FL is considered to contribute to learning impairment.

**Fig 2:**
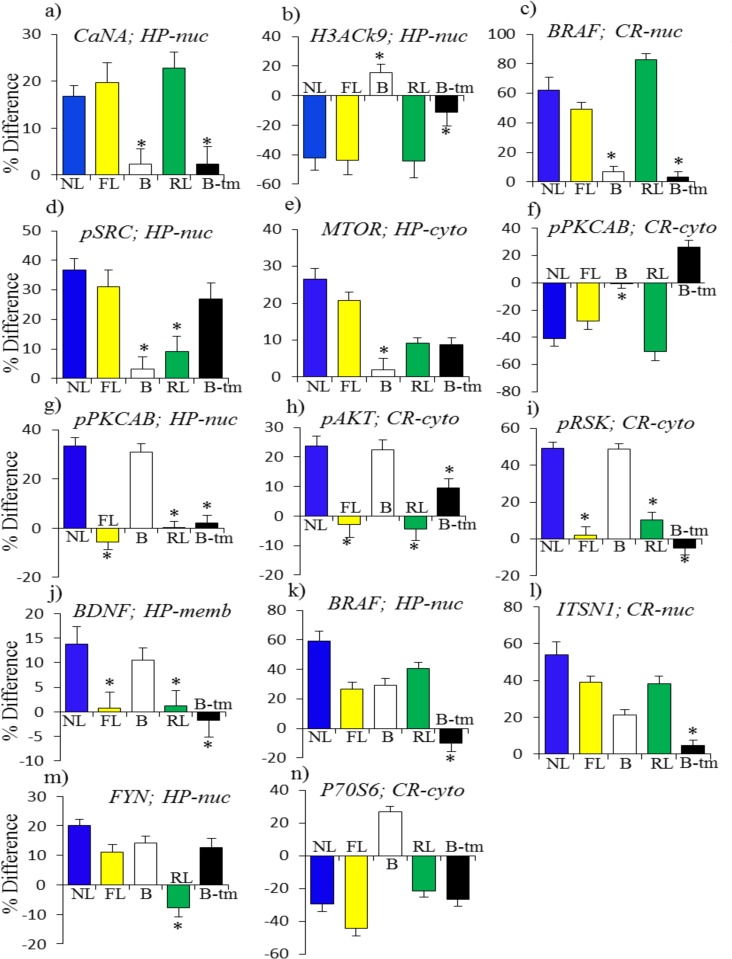
Patterns of protein responses normal in saline-treated Ts65Dn mice: necessary but not sufficient for learning. NL, normal learning, saline treated control mice; FL, failed learning, saline treated Ts65Dn; B, baseline, saline treated Ts65Dn vs saline treated control mice; RL, rescued learning, memantine treated ts65Dn; B-tm, memantine response in Ts65Dn. (a)-(f): FL = NL and RL + B-tm = NL. (g)-(j): FL = 0, B = NL. (k)-(n): FL + B = NL and RL + B-tm + B = NL.

**Fig 3:**
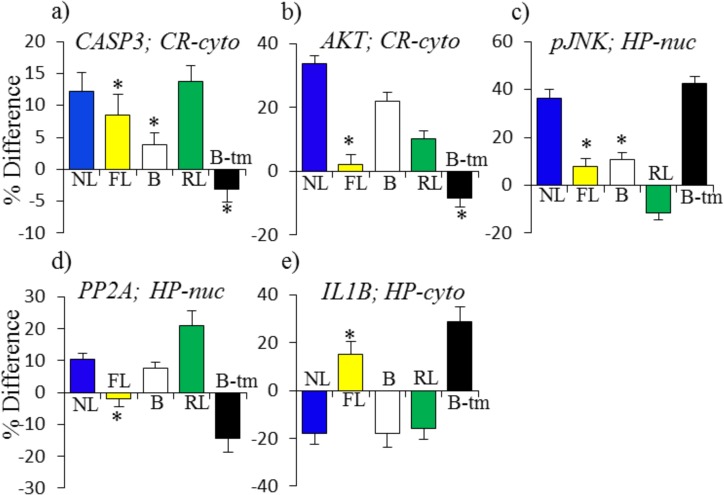
Patterns of protein responses: inadequate in saline treated Ts65Dn mice. FL = 0, and RL + B + B-tm = NL. For abbreviations see Fig. 2.

Tables 4 and 5 list proteins associated with each of these patterns. Instances are separated into those where the magnitudes of responses in failed learning and/or rescued learning are within 10% of those seen in normal learning and those that are similar to controls but differ by >10%. Examples of each pattern are shown in Figs. 2 and 3.

**Table 4:** Protein responses necessary but not sufficient for learning; memantine is not required: NL ≠ 0; B + FL ≈ NL (≤10% difference and >10% difference).

Pattern	Hippocampus		Cortex	
	≤10%	>10%	≤10%	>10%
FL = NL Total: 14 and 10	n: CaNA, H3AcK9	n: pSRC	n: EGR1, H3MeK4, nNOS, SOD1	n: BRAF, pERK, P38
	c: RAPTOR, BAD	c: CDK5, EGR1, pERK, MTOR, pNR1, pNR2A	c:	c: BDNF
	m:NR1, pNR1	m: RCAN1	m:FYN	m: pCAMKII
FL = 0; B = NL Total: 15 and 9	n: JNK, pAKT, pGSK3BS9, pMEK, pPKCAB, RSK	n: ELK, P70S6, PKCA, pCFOS, pMTOR, pRSK	n:	n: CDK5, pNUMB
	c:	c: AKT, P70S6,	c:, pAKT, pNR1, pRSK,	c: ELK, JNK, TIAM1
	m:	m: BDNF	m: pGluR2	m:
FL + B = NL Total: 10 and 4	n: BRAF, FYN	n: H3AcK18, GSK3B	n:DYRK1A, ITSN1	n:
	c: NR2B, pBRAF, pMTOR	c: pMEK	c: P70S6	c: NR2B
	m: NR2A	m: pERK	m:	m:

n, nuclear; c, cytosolic; m, membrane fractions. Total, number of instances in hippocampus and cortex. Other abbreviations as in Table 1.

**Table 5:** Protein responses where memantine is required for learning: NL ≠ 0; FL + B ≠ NL; B + B-tm + RL ≈ NL (≤10% difference and >10% difference).

Pattern	Hippocampus		Cortex	
	≤10%	>10%	≤10%	>10%
RL = NL Total: 5 and 6	n:	n: TRKA	n: AKT	n:
	c:	c: ELK, pNR2B	c: CASP3	c: TRKA, pPKCAB, pBRAF
	m: CDK5	m: CaNA	m: CAMKII	m:
RL = 0; B-tm = NL Total: 1 and 0	n:	n: NUMB	n:	n:
	c:	c:	c:	c:
	m:	m:	m:	m:
B-tm + RL = NL Total: 1and 5	n: pJNK	n:	n: CaNA	n: ERBB4, ubiquitin
	c:	c:	c:	c: GAD2
	m:	m:	m:	m: TIAM1
RL + B = NL Total: 6 and 6	n: pEIF4B, TH	n: pERK	n:	n:
	c:	c: pJNK, ERK	c: AKT	c: CTNNB1, RSK, RCAN1, MTOR
	m: NR2B	m:	m: SYP	m:
RL + B + B-tm = NL Total: 7 and 23	n: Ubiquitin	n: SOD1, ERBB4, PP2A	n: ARC, pS6	n: pPKCAB, IL1B, pGSK3BS9, SNCA
	c: JNK, pP70S6	c: IL1B	c: pMEK, RAPTOR, pNR2A	c: pNUMB, IL1B, S6, pGSK3B-T216, pCASP9, Ubiquitin, pERK, P70S6, BAX, TAU
	m:	m:	m:	m: pERK, TRKA, pNUMB, CTNNB1

n, nuclear; c, cytosolic; m, membrane fractions. Total, number of instances in hippocampus and cortex. Other abbreviations in Table 1.

Fig. 2 shows examples of patterns (i)-(iii). In Fig. 2A, in the nuclear fraction of hippocampus, the level of CaNA increased by 15% in NL and increases of similar magnitudes were also seen in FL and RL. Similarly, a decrease of 40% in the level of H3AcK9 in NL is matched by approximately the same level of decreases in both FL and RL (Fig. 2B). In the nuclear fraction of cortex, levels of BRAF in NL, FL and RL increased between ∼50% and ∼80% (Fig. 2C). In each of these three instances, levels in B were normal and memantine had no effect. However, in a related example from the hippocampus nuclear fraction (Fig. 2D), while levels of pSRC increased by ∼30% in NL and FL, no change was detected in RL. This is likely an example of a requirement for a specific level, and not strictly a change, in protein level, because memantine treatment alone induced an increase of ∼25%, thus obviating the need for a response in RL. Levels of MTOR in the hippocampus cytosol increased by 20% and 25% in NL and FL, but increased by only ∼10% in RL. This is, however, after an increase of ∼10% induced by memantine (Fig. 2E). Thus, in these cases, while memantine was not required for an appropriate response (because FL = NL), it can alter responses in RL both directly and indirectly. As a last example of a pattern when FL = NL, for pPKCAB in cortex cytosol, the decreases in NL and FL of 30%–40% are exceeded in RL by a decrease of ∼50% (Fig. 2F). This is however relative to the ∼25% increase induced by memantine, thus again achieving a similar outcome in protein level. Table 4 lists the 14 instances in hippocampus and the ten in cortex where responses in FL are normal, i.e. equivalent to NL.

In Fig. 2G-J, instances are shown where there are no changes in FL or RL, and the abnormal levels in B are of an appropriate magnitude to compensate for changes occurring in NL. These instances include a 30% increase in pPKCAB in the nuclear fraction of the hippocampus, increases of 20% in the level of pAKT and ∼50% in pRSK in the cortex cytosol, and ∼10% in BNDF in the hippocampus membrane fraction. Table 4 lists totals of 15 and nine instances in hippocampus and cortex respectively where levels in B are approximately equal to those after NL.

Fig. 2K-N illustrate instances in which the levels of the abnormalities in B only partially compensate for changes in NL levels, with the result that the magnitudes of the changes in FL and RL are less than those in NL. For BRAF in the hippocampus nuclear fraction, the increase in NL of ∼60% is achieved by a baseline level of ∼30% plus increases in both FL and RL of ∼30%. Similarly for ITSN1 in the nuclear fraction of cortex, increases in FL and RL of ∼35% added to the elevated levels in B of ∼20% can achieve a level similar to the increase of 55% in NL. For Fyn in the nuclear fraction of hippocampus, the lack of a response in RL is compensated by an increase in B-tm, so that levels in B plus changes in FL and levels in B plus changes in B-tm are both equivalent to the increase in NL. As a slightly different example, the decrease of 30% in the level of P70S6 in NL, seen in the cytosolic fraction of cortex, is achieved in FL by a larger decrease of 45% that also compensates for the initial elevated level in B; a more modest decrease in RL is compensated by the prior decrease induced by memantine. Thus, levels of both B + FL and B + B-tm + RL are equivalent to changes in NL. There are ten and four instances of these types of patterns in hippocampus and cortex, respectively.

Fig. 3 shows examples of instances where responses seen in NL are absent in FL and are not compensated, or not sufficiently compensated, by B. Memantine produces either direct or indirect changes in protein levels such that B-tm + RL is similar to NL, suggesting that the lack of responses in FL likely contribute to learning impairment in untreated Ts65Dn. Indirect responses to memantine in RL include, in the cytosolic fraction of cortex, an increase in the level of CASP3 similar to the ∼12% levels seen in NL (Fig. 3A) and an increase in the level of AKT that, added to levels in B, approximates the increase in NL (Fig. 3B). A direct effect of memantine is seen in the increase of ∼40% in levels of pJNK in the nuclear fraction of hippocampus; this matches the increase in NL and no further change occurs in RL (Fig. 3C). More complex responses to memantine are seen in the responses of PP2A in the nuclear fraction and IL1B in the cytosolic fraction of hippocampus (Fig. 3D,E). In both of these cases, the direction of memantine-induced changes (B-tm) is opposite to that in NL. These may therefore be examples of a protein level not being adequate, even when it is similar to the level reached in NL. Instead, a change in level is required and this is facilitated by first correcting the abnormal level in B. In hippocampus, there are 20 instances of responses in NL absent in FL being produced by memantine, and 40 in cortex (Table 5).

**Fig 4:**
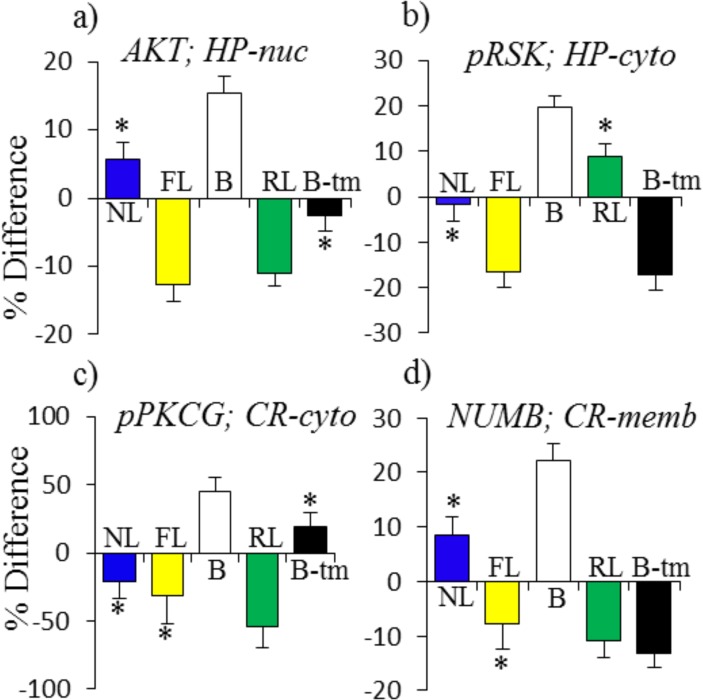
Patterns of protein responses: abnormal in baseline and NL = 0. **(a), (b), normalized in FL and RL + B-tm; (c), (d), normalized in RL + B-tm only.** For abbreviations see Fig. 2.

Additional patterns involve proteins that do not change in NL but that are abnormal in B. As shown in Fig. 4, some of the abnormalities in B are compensated by changes in both FL and RL or in FL and in B-tm (e.g. AKT and pRSK in the hippocampus nuclear and cytosolic fraction, respectively (Fig. 4A,B)). In other cases, compensatory responses do not occur in FL, but are induced indirectly in RL, e.g. pPKCG in the cortex cytosol, or directly by memantine, e.g. NUMB in the cortex membrane fraction (Fig. 4C,D). The data in Fig. 4C,D again suggest that these abnormalities in B may contribute to learning impairment in untreated Ts65Dn.

Other abnormalities in B, however, are not compensated in RL or FL or by B-tm. In hippocampus, there are 11 such instances (six in the nuclear fraction, four in the cytosolic and one in the membrane), and interestingly seven are Hsa21-encoded proteins (discussed further below).

### Hsa21 proteins

**Fig 5:**
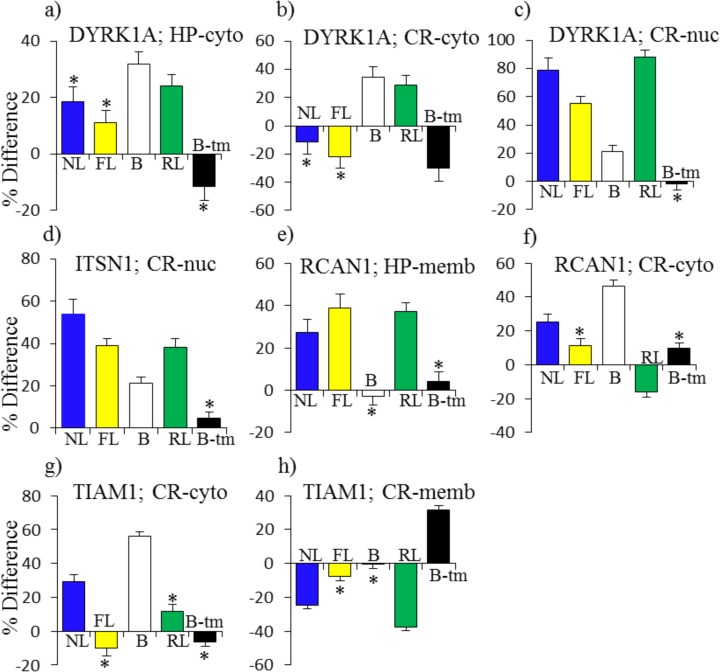
Patterns of protein responses: Hsa21-encoded proteins. For abbreviations see Fig. 2.

Levels of seven Hsa21 proteins were analyzed in the nuclear and cytosolic fractions and five in the membrane fractions. As has been seen in studies at the RNA level [44], levels of trisomic proteins were not elevated in all fractions. ITSN1 was the only one elevated in all three fractions of the hippocampus, and no protein was elevated in all fractions of the cortex. APP and ITSN1 were both elevated in the membrane fraction of both hippocampus and cortex. In Fig. 5, selected instances of two of the most popular Hsa21 proteins, DYRK1A and RCAN1, plus ITSN1 and TIAM1, are shown. Each of these proteins interacts with or indirectly impacts the activity of the NMDAR and each responds in NL in at least one fraction-brain region.

DYRK1A is a protein kinase extensively studied for causing learning/memory impairment when overexpressed. It is implicated in impairment in the Ts65Dn because both its inhibition with EGCG and its genetic reduction to disomy rescue at least some of the L/M abnormalities [44,45]. DYRK1A is elevated in B in the cytosolic fraction of hippocampus and in the cytosolic and nuclear fractions of cortex (Fig. 5A-C). Memantine corrects the abnormally high level only in cortex cytosol, and in spite of elevated initial abnormalities, levels of DYRK1A further increase in RL in two fractions. The increase in levels of DYRK1A are particularly robust in the nuclear fraction of cortex, at ∼80% in NL, 50% in FL and ∼90% in RL. Together these data show that successful learning occurs in the presence of abnormal levels of DYRK1A.

In the nuclear fraction of cortex, the multi-domain endocytosis protein ITSN1 was elevated in B, and increased even further in both FL and RL, matching the ∼50% increase seen in NL (Fig. 5D). In hippocampus, levels of ITSN1 were elevated and not affected by memantine with the exception of an indirect decrease seen in RL in the membrane fraction (not shown). Interestingly, NL is associated with increases in the calcineurin modulator RCAN1 in the membrane fraction of hippocampus and in the cytosolic fraction of cortex (Fig. 5E,F). These increases were matched in FL and RL in hippocampus. However, in cortex the baseline elevation of RCAN1 was modulated by a decrease in RL, while no responses occurred in FL. Lastly, the guanine nucleotide exchange protein, TIAM1, that functions in part by interacting with NMDA receptors at the membrane, displays interesting reciprocal responses. In NL, in cortex, TIAM1 levels increased in the cytosol, while they decreased in the membrane (Fig. 5G,H). In untreated Ts65Dn, the former change may be compensated by the elevated level in the cytosol in B, but in the membrane, memantine induces an increase followed by a decrease in RL. The lack of any response in FL thus may contribute to learning impairment in untreated Ts65Dn.

Together these data indicate not only that successful learning occurs in the presence of abnormally high levels of several Hsa21 proteins, but also that changes in these proteins are associated with normal learning and that baseline high levels do not impede their further increase in FL and RL.

### NMDAR subunits

**Fig 6:**
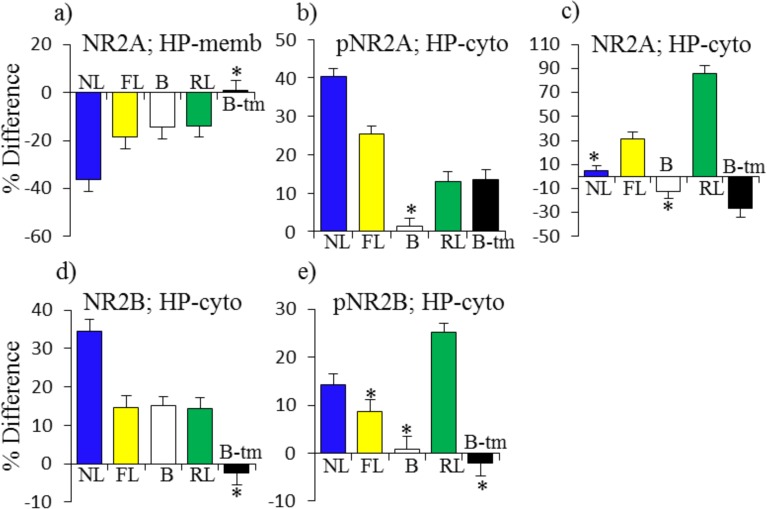
Pattern of protein responses: NMDAR subunits in hippocampus. For abbreviations see Fig. 2.

We next examined patterns in subunits of the NMDAR in response to learning and memantine. Although it binds to NR2A and NR2B to inhibit NMDAR activity and dynamics, in hippocampus, memantine directly induces few changes in levels, phosphorylation or localization of NMDAR subunits (Fig. 6A-E). For NR2A, decreases in the membrane fraction of 35% in NL are compensated and matched by decreases of 20% in FL added to the initial repressed level of 15% in B. Memantine does not affect this response because the change in RL is approximately equal to that in FL (Fig. 6A). For pNR2A, the increase of ∼40% in the cytosol in NL is partially compensated by increases in FL of ∼25% and increases in B-tm and RL of ∼13% each (Fig. 6B). Memantine also affects cytosolic levels of NR2A. It first induces a decrease of ∼30% which is followed by an increase of ∼80% in RL. This is a uniquely strong response to memantine, because there is no change in NL and only a modest increase of 30% in FL (Fig. 6C). Patterns of NR2B responses are different. The increase in cytosolic NR2B in NL of ∼30% is matched by the elevated level in B plus the increases in both FL and RL (Fig. 6D). Levels of pNR2B increase in both NL and RL, but not FL (Fig. 6E).

**Fig 7:**
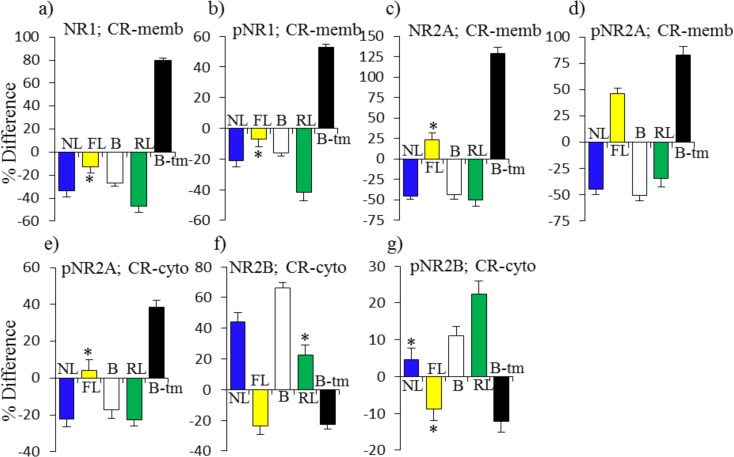
Patterns of protein responses: NMDAR subunits in cortex. For abbreviations see Fig. 2.

In cortex, direct effects of memantine are much more dramatic (Fig. 7A-G). In the membrane fraction, memantine treatment alone induces increases of >50%–100% in NR1, pNR1, NR2A and pNR2A (Fig. 7A-D). This is in contrast to decreases in each of these in NL. Interestingly, the repressed levels in B match the decreases in NL. The memantine responses, that include subsequent decreases also in RL, therefore suggest that dynamic responses are required for successful learning, and static levels are inadequate. There are no responses in FL, with the exception of an increase in pNR2A, further suggesting that impaired learning at least in part is related to failed and abnormal responses in NMDAR subunits. Cytosolic responses in pNR2A and NR2B seen in NL are absent or in the opposite direction in FL (Fig. 7E-G)

## Discussion

The Ts65Dn display many features relevant to DS, including impaired performance in L/M tasks, such as the Morris water maze, object recognition and CFC, that require a functional hippocampus [10]. The Ts65Dn mice are trisomic for orthologs of 88 Hsa21 and 50 non-Hsa21 protein coding genes. Available information on the functions of these genes, although limited overall, clearly shows that they impact many pathways and cellular processes involved in brain development and function [5, 13, 46]. When they are overexpressed, it is reasonable to expect that the molecular consequences will propagate in complex and unpredictable directions, affecting multiple pathways, many cellular processes, and certainly extending to non-trisomic genes. Such predictions have been proven correct in the many gene expression experiments that have been carried out at the RNA level [44]. Although Hsa21 orthologs that have been studied intensively, such as DYRK1A, RCAN1 and APP, clearly have functions that may contribute, when they are overexpressed in trisomy, to learning/memory and/or brain development, structure and function, the remaining Hsa21 genes cannot be eliminated from possible phenotypic contributions. However, even with knowledge of the functions of individual genes, the consequences of their concomitant overexpression in full trisomy Hsa21, where functional interactions among trisomic genes will occur, will not be known. Thus, instead of a focus on expression patterns of Hsa21 orthologs, we continue to pursue a broad view of how trisomy in the Ts65Dn mouse model of DS propagates to non trisomic genes to perturb pathways and processes relevant to learning.

RPPA has the advantage of facilitating measurement of a larger number of proteins in a larger number of samples than is practical by western blots and immunohistochemistry. Here we assayed expression levels of 85 proteins/protein modifications in six brain region/subcellular fractions from 40 Ts65Dn and compared them to those previously reported for their littermate controls. The sensitivity of RPPA and the measurement of signal intensities in three replicates of a five point dilution series from each sample allows the determination of highly accurate mean values for each group of samples [43]. As a result, a large number of significant differences were detected between trisomics and controls, between saline vs. memantine treatment and between CS vs SC groups (Table 2). This is so in spite of the use of the Bonferroni correction for multiple testing, which is a conservative method for controlling the number of false positives (although causing a higher proportion of false negatives) [47].

Given the number of changes and differences between groups, it is a challenge to evaluate their individual and aggregate importance. As shown in Table 2, in NL a total of 90 changes (67 increases and 23 decreases) in protein levels occurred in hippocampus and 80 in cortex (42 increases and 38 decreases). A first interesting point is that even though saline injected Ts65Dn fail to learn in CFC, a total of 39 of the 90 responses seen in hippocampus can be judged as adequate in FL (Table 4). This judgment is based on FL responses fulfilling one of the following criteria: the magnitude of the response in FL is similar to that in NL (14 proteins), an abnormal protein level at baseline in the Ts65Dn is similar in magnitude to the change in NL and therefore can compensate for the response in NL (15 proteins), or the sum of a baseline abnormality plus a smaller change in FL is similar in magnitude to the change in NL (ten proteins). As is clear from Table 4, these apparently adequate changes in FL are not exclusive or restricted to any specific pathway or cellular process. Proteins include MAPK components BRAF, pBRAF, pMEK, pERK, pRSK and ELK, and MTOR components MTOR, pMTOR, RAPTOR and P70S6, as well as cytosolic levels of the immediate early gene protein EGR1 and nuclear levels of pCFOS. Nuclear levels/changes of the histone modifications, H3AcK9 and H3AcK18, and membrane levels of BDNF were also adequate in FL. All of these proteins have well established roles in L/M and their responses in NL are reasonable [39]. Additional support for the requirement of these responses for successful learning is the observation that, in each case, similar responses are present in RL with or without modulation by memantine. Thus, these responses can be considered necessary for successful learning, but clearly, by themselves, they are not sufficient.

A second set of 20 responses observed in hippocampus in NL were either absent or inadequate in FL, but were rescued with memantine treatment, either directly or indirectly. Direct rescue is concluded when the response in B-tm alone or B-tm added to a perturbed level in B is similar to that seen in NL. In other instances, memantine either indirectly induced a response in RL similar to that in NL, or combined with an abnormal level in B to indirectly result in a similar change. The 20 proteins requiring memantine to induce an NL-like response include nuclear levels of pERK and cytosolic levels of ELK, as well as cytosolic levels of the MTOR pathway component pP70S6 and nuclear levels of the downstream target, pEIF4B. Changes in JNK and pJNK were also found in this group.

Of the 80 responses observed in cortex in NL, 23 occurred in saline injected Ts65Dn and a further 40 were directly or indirectly induced following memantine treatment. The nature and distribution of proteins was different from those in hippocampus, but still included components of MAPK and MTOR pathways. Notably, even when the same proteins were affected in hippocampus and cortex, the requirement for memantine was not necessarily the same. For example, increases in cytosolic levels of pERK, MTOR and RAPTOR and nuclear levels of pPKCAB occurred without memantine in hippocampus, but required memantine in cortex. Conversely, increases in nuclear levels of pERK and cytosolic levels of JNK required memantine in hippocampus but not in cortex.

Thus, in total, of 90 responses occurring in NL in hippocampus, 59 (∼66%) occurred in FL and/or RL, and of 80 in cortex, 63 (80%) occurred in FL and/or RL.

Levels of seven Hsa21 proteins were measured in nuclear and cytosolic fractions and four in membrane fractions. Each was abnormal in at least one fraction of hippocampus and/or cortex. With the exception of levels of DYRK1A in the cortex cytosolic fraction, memantine treatment did not normalize the elevated levels. It is of interest that successful learning can therefore occur in the presence of elevated levels of these proteins. Furthermore, it is also interesting that the Hsa21 proteins, DYRK1A, ITSN1, RCAN1 and TIAM1, also respond in control mice in normal successful learning in CFC.

The responses of NMDAR subunits were perhaps unanticipated. In hippocampus, in FL, most responses appeared to be adequate, either similar to those in NL or partially compensated by elevated levels in B. Unique, however, in RL in hippocampus, is the ∼80% increase in NR2A levels in the cytoplasm. In cortex, in particular in the membrane, phosphorylated and total forms of NR1 and NR2A all increased, in some cases by >80%-125%, among the most robust responses seen in these experiments. These increases are in contrast to the uniform decreases in the same proteins in NL, often by ∼50%. Notably, each of these proteins showed repressed levels in B, and generally failed to respond in FL. It is possible that these are additional instances where dynamic responses are required and abnormal static levels cannot compensate. The strong increases directly induced by memantine can be interpreted as facilitating the subsequent correct dynamic responses that are then seen in RL. While hippocampus has most often been studied with respect to CFC, prefrontal cortex has also been implicated [48], but details of associated protein responses have not previously been described.

As a last consideration, it is noteworthy that the large numbers of initial abnormalities in B are significantly reduced after CFC in the presence of memantine, i.e. when protein levels in RL are compared to those in NL (Table 2, last column). For example, in the nuclear fraction of hippocampus, the initial 41 elevated protein levels in B are reduced to only five, of which three are Hsa21 proteins. Similarly in the cytosolic fraction of cortex, the initial abnormalities in B of 30 elevated and 12 repressed are reduced to 10 elevated (of which four are Hsa21 proteins) and only four are repressed. Thus, the abnormalities in B and in FL are largely redressed by direct and indirect effects of memantine.

The limitations of the current approach and datasets must be considered if the results are to best guide future experiments. Experiments were limited to examination of a single time point, 60 minutes, post training in CFC. Although results in NL are consistent with previous reports from the literature [39], the data present a snap shot of responses to the stimulation of CFC learning. Importantly, information is lacking on the time course of responses, i.e. when the Ts65Dn show abnormalities in FL, we do not know if this is observed because the responses are indeed different in magnitude from controls, or if instead the normal responses are occurring more slowly, and have not yet peaked, or more quickly and have already peaked, and are now returning to initial levels. Such perturbations in timing in FL could be responsible for downstream perturbations in the required dynamics of signaling processes, perturbations that cannot be overcome by the proportion of normal responses that occur in FL.

Responses to a single drug were examined here. More than 20 drugs/small molecules have now been shown to rescue or partially rescue L/M in one or more tasks in the Ts65Dn (and 5 have failed) [13]. Given the diversity in the targets and mechanisms of action of these drugs, insight would be obtained by determining common and differential protein responses among these drugs in CFC and in other L/M tasks. Importantly, not all successful drugs rescued L/M in all tasks assayed, e.g. lithium and a GABAB antagonist failed to rescue performance in spontaneous alternation tasks although they succeeded in at least partially rescuing impairments in CFC [49,50], and GABAA antagonists failed to rescue retention in the Morris water maze although they rescued acquisition [14,15]. Improved understanding of L/M at the protein level could lead to development of effective combinations of drugs, or to identification of individual drugs that more effectively rescue performance in multiple L/M tasks. Given the genetic limitations of the Ts65Dn as a model for DS, it is also important to extend these experiments to other mouse models, trisomic for different subsets of Hsa21 orthologs, to more accurately predict if the molecular basis of drug responses seen in the Ts65Dn is likely to be recapitulated in full trisomy of human DS.

Lastly, it must be emphasized that the data here were generated from male mice. Significant gender differences in patterns of gene expression have been demonstrated at the RNA level [51] and preliminary studies in mouse models of DS indicate extensive gender differences at the protein and protein modification levels (Block and Gardiner, unpublished). Among the reports of L/M rescue in the Ts65Dn are data to indicate that, at least for some drugs, age at treatment affects outcome [17]. Further studies therefore will also be required to determine how age influences observations of both baseline perturbations and drug responses.

## Supporting Information

S1 Table Mouse information: littermates; tissue weights. (DOC)

S2 Table Antibody information: common and official protein names; antibody source, catalogue number and dilution. (DOC)

S3 Table Protein differences between genotypes and protein changes with treatments, nine pairwise comparisons. (XLS)

S1 Text Additional protein patterns: stable proteins and proteins responding in a single comparison. (DOCX)
