# Supplementary material for: Protein Dynamics Associated with Failed and Rescued Learning in the Ts65Dn Mouse Model of Down Syndrome
Source: PLoS One. 2015 Mar 20;10(3):e0119491. doi: 10.1371/journal.pone.0119491 (PMC4368539; doi:10.1371/journal.pone.0119491)
Supplement: S1 Table — (DOC) [file pone.0119491.s001.doc]

**Supplementary Table S1. Information for individual mice.** Age at sacrifice (months), weight of each brain region (mg), NA-not available, SC, shock-context; CS, context-shock. Littermates are housed together. HP, hippocampus; CR, cortex.

| Mouse | Littermates | Age (m) | Genotype | Behavior | Treatment | HP | CR |
| --- | --- | --- | --- | --- | --- | --- | --- |
| 1 | 41,42 | 3.5 | Control | SC | saline | 23 | 161 |
| 2 |  | 4 | Control | SC | saline | 25 | 170 |
| 3 | 4,43,44,45 | 4 | Control | SC | saline | 24 | 143 |
| 4 | 3,43,44,45 | 4 | Control | SC | saline | 38 | 170 |
| 5 | 17,18,19,20,59 | 4 | Control | SC | saline | 29 | 140 |
| 6 | 7,8,9,46,47 | 4 | Control | SC | saline | 28 | 175 |
| 7 | 6,8,9,46,47 | 4 | Control | SC | saline | 31 | 172 |
| 8 | 6,7,9,46,47 | 4 | Control | SC | saline | 38 | 160 |
| 9 | 6,7,8,46,47 | 4 | Control | SC | saline | 23 | NA |
| 10 | 48,49 | 3 | Control | SC | saline | 34 | 150 |
| 11 | 12,13 | 3 | Control | CS | saline | 32 | NA |
| 12 | 11,13 | 3 | Control | CS | saline | 35 | NA |
| 13 | 11,12 | 3 | Control | CS | saline | 31 | NA |
| 14 | 15,16,56,57,58 | 4 | Control | CS | saline | 43 | NA |
| 15 | 14,16,56,57,58 | 4 | Control | CS | saline | 33 | NA |
| 16 | 14,15,56,57,58 | 4 | Control | CS | saline | 27 | NA |
| 17 | 5,18,19,20,59 | 4 | Control | CS | saline | 36 | NA |
| 18 | 5,17,19,20,59 | 4 | Control | CS | saline | 33 | NA |
| 19 | 5,17,18,20,59 | 4 | Control | CS | saline | 28 | NA |
| 20 | 5,17,18,19,59 | 4 | Control | CS | saline | 35 | NA |
| 21 | 60,61 | 3.5 | Control | SC | memantine | 22 | 170 |
| 22 | 23 | 3 | Control | SC | memantine | 35 | 145 |
| 23 | 22 | 3 | Control | SC | memantine | 24 | 140 |
| 24 | 31 | 3 | Control | SC | memantine | 33 | 130 |
| 25 | 26,27.28,62,63 | 3 | Control | SC | memantine | 41 | 126 |
| 26 | 25,27,28,62,63 | 3 | Control | SC | memantine | 31 | 160 |
| 27 | 25,26,28,62,63 | 3 | Control | SC | memantine | 38 | 150 |
| 28 | 25,26,27,62,63 | 3 | Control | SC | memnatine | 39 | 150 |
| 29 | 30,64,65 | 3 | Control | SC | memantine | 33 | 152 |
| 30 | 29,64,65 | 3 | Control | SC | memantine | 41 | 153 |
| 31 | 24 | 3 | Control | CS | memantine | 38 | NA |
| 32 | 70 | 3.5 | Control | CS | memantine | 44 | NA |
| 33 | 34,35,71,72 | 3 | Control | CS | memantine | 41 | NA |
| 34 | 33,35,71,72 | 3 | Control | CS | memantine | 31 | NA |
| 35 | 33,34,71,72 | 3 | Control | CS | memantine | 30 | NA |
| 36 | 73 | 3 | Control | CS | memantine | 26 | NA |
| 37 | 38 | 3 | Control | CS | memantine | 48 | NA |
| 38 | 37 | 3 | Control | CS | memantine | 51 | NA |
| 39 | 40,66,67,77,78 | 3 | Control | CS | memantine | 40 | NA |
| 40 | 39,66,67,77,78 | 3 | Control | CS | memantine | 36 | NA |
| 41 | 1,42, | 3.5 | Ts65Dn | SC | saline | 24 | 150 |
| 42 | 1,41 | 3.5 | Ts65Dn | SC | saline | 26 | 169 |
| 43 | 3,4,44,45 | 4 | Ts65Dn | SC | saline | 40 | 173 |
| 44 | 3,4,43,45 | 4 | Ts65Dn | SC | saline | 23 | 182 |
| 45 | 3,4,43,44 | 4 | Ts65Dn | SC | saline | 29 | 192 |
| 46 | 6,7,8,9,47 | 4 | Ts65Dn | SC | saline | 21 | 200 |
| 47 | 6,7,8,9,46 | 4 | Ts65Dn | SC | saline | 25 | 165 |
| 48 | 10,49 | 3 | Ts65Dn | SC | saline | 31 | 200 |
| 49 | 10,48 | 3 | Ts65Dn | SC | saline | 27 | 179 |
| 50 | 51,52 | 3.5 | Ts65Dn | CS | saline | NA | NA |
| 51 | 50,52 | 3.5 | Ts65Dn | CS | saline | NA | NA |
| 52 | 50,51 | 3.5 | Ts65Dn | CS | saline | NA | NA |
| 53 | 54,55 | 3 | Ts65Dn | CS | saline | NA | NA |
| 54 | 53,55 | 3 | Ts65Dn | CS | saline | NA | NA |
| 55 | 53,54 | 3 | Ts65Dn | CS | saline | NA | NA |
| 56 | 14,15,16,57,58 | 4 | Ts65Dn | CS | saline | NA | NA |
| 57 | 14,15,16,56,58 | 4 | Ts65Dn | CS | saline | NA | NA |
| 58 | 14,15,16,56,57 | 4 | Ts65Dn | CS | saline | NA | NA |
| 59 | 5,17,18,19,20 | 4 | Ts65Dn | CS | saline | NA | NA |
| 60 | 21,61 | 3.5 | Ts65Dn | SC | memantine | 22 | 170 |
| 61 | 21,60 | 3.5 | Ts65Dn | SC | memantine | 34 | NA |
| 62 | 25,26,27,28,63 | 3 | Ts65Dn | SC | memantine | 39 | 190 |
| 63 | 25,26,27,28,62 | 3 | Ts65Dn | SC | memantine | 45 | 135 |
| 64 | 29,30,65 | 3 | Ts65Dn | SC | memantine | 37 | 160 |
| 65 | 29,30,64 | 3 | Ts65Dn | SC | memantine | 44 | 190 |
| 66 | 39,40,67,77,78 | 3 | Ts65Dn | SC | memantine | 36 | 170 |
| 67 | 39,40,66,77,78 | 3 | Ts65Dn | SC | memantine | 36 | 150 |
| 68 | 69 | 2.5 | Ts65Dn | SC | memantine | 44 | 160 |
| 69 | 68 | 2.5 | Ts65Dn | SC | memantine | 44 | 145 |
| 70 | 32 | 3.5 | Ts65Dn | CS | memantine | NA | NA |
| 71 | 33,34,35,72 | 3 | Ts65Dn | CS | memantine | NA | NA |
| 72 | 33,34,35,71 | 3 | Ts65Dn | CS | memantine | NA | NA |
| 73 | 36 | 3 | Ts65Dn | CS | memantine | NA | NA |
| 74 | 75,76 | 3 | Ts65Dn | CS | memantine | NA | NA |
| 75 | 74,76 | 3 | Ts65Dn | CS | memantine | NA | NA |
| 76 | 74,75 | 3 | Ts65Dn | CS | memantine | NA | NA |
| 77 | 39,40,66,67,78 | 3 | Ts65Dn | CS | memantine | NA | NA |
| 78 | 39,40,66,67,77 | 3 | Ts65Dn | CS | memantine | NA | NA |
| 79 | 37,38 | 3 | Ts65Dn | CS | memantine | NA | NA |
