# Supplementary material for: Protein Dynamics Associated with Failed and Rescued Learning in the Ts65Dn Mouse Model of Down Syndrome
Source: PLoS One. 2015 Mar 20;10(3):e0119491. doi: 10.1371/journal.pone.0119491 (PMC4368539; doi:10.1371/journal.pone.0119491)
Supplement: S2 Table — (DOC) [file pone.0119491.s002.doc]

**Supplementary Table S2. Protein and antibody information.** All proteins used in RPPA are listed. Functional class: ID, mutation in the human gene results in intellectual disability (Sturgeon et al 2012). LM, SP and ST: mutation of the mouse gene results in learning/memory, synaptic plasticity and synaptic transmission, respectively (information from the Mammalian Phenotype Browser, <http://www.informatics.jax.org/searches/MP_form.shtml>)

| **Common protein name** | **Official name** | **Functional class** | | | | **Antibody Source** | **Catalogue #** | **Dilution** |
| --- | --- | --- | --- | --- | --- | --- | --- | --- |
|  |  | **ID** | **LM** | **SP** | **ST** |  |  |  |
| AKT | AKT1 |  |  |  | Y | Santa Cruz Biotechnology | SC-1619 | 1:300 |
| AMPKA | PRKAA1 |  |  |  |  | Cell Signaling | 2532 | 1:750 |
| APP | APP |  | Y |  | Y | Cell Signaling | 2452 | 1:300 |
| ARC | ARC |  | Y |  | Y | Abcam | Ab118929 | 1:300 |
| BAD | BAD |  |  |  |  | Cell Signaling | 9292 | 1:500 |
| BAX | BAX |  |  |  |  | Cell Signaling | 2772 | 1:750 |
| BCL2 | BCL2 |  |  |  |  | Cell Signaling | 2870 | 1:500 |
| BDNF | BDNF |  | Y | Y | Y | Epitomics | 2960-1 | 1:500 |
| BRAF | BRAF | Y | Y |  | Y | Cell Signaling | 9434 | 1:500 |
| CAMKII | CAMK2 |  | Y | Y | Y | Cell Signaling | 3362 | 1:500 |
| CaNA | PPP3CA |  |  |  |  | Cell Signaling | 2614 | 1:1000 |
| CASP3 | CASP3 |  | Y | Y | Y | Cell Signaling | 9662 | 1:500 |
| CDK5 | CDK5 |  | Y |  | Y | Cell Signaling | 2506 | 1:500 |
| cFOS | CFOS |  | Y |  | Y | Cell Signaling | 2250 | 1:300 |
| CREB | CREB1 |  | Y |  | Y | Cell Signaling | 9197 | 1:500 |
| DYRK1A | DYRK1A | Y |  |  |  | Abnova | H00001859-M01 | 1:750 |
| EGR1 | EGR1 |  | Y |  |  | Cell Signaling | 4154 | 1:300 |
| ELK1 | ELK1 |  |  |  |  | Santa Cruz Biotechnology | Sc-355 | 1:300 |
| ERBB4 | ERBB4 |  | Y |  | Y | Protein Technology | 19943-1-AP | 1:300 |
| ERK1/2 | MAPK1/3 |  | Y |  | Y | Santa Cruz Biotechnology | SC-153 | 1:300 |
| FYN | FYN |  | Y |  | Y | Cell Signaling | 4023 | 1:500 |
| GAD2 | GAD2 |  |  |  |  | Cell Signaling | 3988 | 1:1000 |
| GFAP | GFAP | Y | Y |  | Y | Epitomics | 2301-1 | 1:500 |
| GluR3 | GRIA3 | Y |  |  | Y | Cell Signaling | 5117 | 1:750 |
| GluR4 | GRIA4 |  | Y |  | Y | Cell Signaling | 3824 | 1:300 |
| GSK3B | GSK3B |  | Y |  | Y | BD Biosciences | 610201 | 1:3000 |
| Histone H3 (AcK18) | HIST3H3 | Y |  |  |  | Cell Signaling | 9675 | 1:300 |
| Histone H3 (AcK9) | HIST3H3 | Y |  |  |  | Cell Signaling | 9649 | 1:300 |
| Histone H3 (DMeK4) | HIST3H3 | Y |  |  |  | Cell Signaling | 9725 | 1:300 |
| IL1B | IL1B |  |  |  |  | Abnova | H00003553 | 1:3000 |
| ITSN1 | ITSN1 |  |  |  |  | BD Biosciences | 611574 | 1:1500 |
| JNK | MAPK8 |  |  |  |  | Cell Signaling | 9252 | 1:500 |
| MEK1/2 | MAP2K1/2 | Y |  |  |  | Cell Signaling | 9122 | 1:500 |
| MTOR | MTOR |  |  |  |  | Cell Signaling | 2972 | 1:500 |
| nNOS | NOS1 |  | Y |  | Y | Cell Signaling | 4234 | 1:500 |
| NR1 | GRIN1 | Y | Y | Y | Y | Upstate Biotechnology | 07-362 | 1:500 |
| NR2A | GRIN2A | Y | Y |  | Y | PhosphoSolutions | 1497-NR2A | 1:500 |
| NR2B | GRIN2B | Y | Y | Y | Y | PhosphoSolutions | 1498-NR2B | 1:500 |
| NUMB | NUMB |  |  |  |  | Cell Signaling | 2756 | 1:500 |
| P35/25 | CDK5R1 |  | Y |  | Y | Cell Signaling | 2680 | 1:750 |
| P38 | MAPK14 |  |  |  |  | Cell Signaling | 9212 | 1:500 |
| P70S6 | RPS6KB1 |  |  |  |  | Santa Cruz Biotechnology | SC-8418 | 1:400 |
| pAKT (Ser473) | AKT1 |  |  |  | Y | Cell Signaling | 4060 | 1:500 |
| pBRAF(Thr401) | BRAF | Y | Y |  | Y | Epitomics | 2298-1 | 1:1000 |
| pCAMKIIA/B( Thr286) | CAMK2A/B |  | Y | Y | Y | PhosphoSolutions | p1005-286 | 1:4000 |
| pCASP9(Ser196) | CAPS9 |  |  |  |  | Abgent | AP3044a | 1:300 |
| pcFOS | CFOS |  | Y |  | Y | Cell Signaling | 5348 | 1:300 |
| pCREB( Ser133) | CREB1 |  | Y |  | Y | PhosphoSolutions | p1010-133 | 1:500 |
| pEIF4B(Ser422) | EIF4B |  |  |  |  | Cell Signaling | 3591 | 1:500 |
| pELK1 (Ser383) | ELK1 |  |  |  |  | Santa Cruz Biotechnology | SC-8406 | 1:500 |
| pERK1/2(Tyr204) | MAPK1/3 |  | Y |  | Y | Santa Cruz Biotechnology | SC-7383 | 1:500 |
| pGluR2(Tyr876) | GRIA2 |  | Y | Y | Y | Cell Signaling | 4027 | 1:500 |
| pGSK3B(Ser9) | GSK3B |  | Y |  | Y | Cell Signaling | 9323 | 1:750 |
| pGSK3B (Tyr216) | GSK3B |  | Y |  | Y | BD Biosciences | 612312 | 1:2000 |
| pJNK(Thr183/Tyr185) | MAPK8 |  |  |  |  | Cell Signaling | 9251 | 1:1000 |
| PKCA | PRKCA |  |  |  |  | Cell Signaling | 2056 | 1:500 |
| pMEK1/2(Ser217/221) | MAP2K1/2 | Y |  |  |  | Cell Signaling | 9154 | 1:750 |
| pMTOR(Ser2448) | MTOR |  |  |  |  | Cell Signaling | 2971 | 1:500 |
| pNR1(Ser889) | GRIN1 | Y | Y | Y | Y | Epitomics | 2329-1 | 1:500 |
| pNR2A(Tyr1246) | GRIN2A | Y | Y |  | Y | Cell Signaling | 4206 | 1:500 |
| pNR2B(Tyr1336) | GRIN2B | Y | Y | Y | Y | PhosphoSolutions | p1516-1336 | 1:500 |
| pNUMB(Ser276) | NUMB |  |  |  |  | Cell Signaling | 4140 | 1:500 |
| PP2A | PPP2R1A |  |  |  |  | Cell Signaling | 2041 | 1:750 |
| pP70S6(Thr389) | RPS6KB1 |  |  |  |  | Cell Signaling | 9205 | 1:500 |
| pPKCA/B(Thr638/641) | PRKAA/B |  |  |  |  | Cell Signaling | 9375 | 1:750 |
| pPKCG (Thr514) | PRKCAG | Y | Y |  | Y | Cell Signaling | 9379 | 1:750 |
| pRSK(Ser380) | RPS6KA3 | Y |  |  |  | Cell Signaling | 9341 | 1:500 |
| pS6(Ser240/244) | RPS6 |  |  |  |  | Cell Signaling | 5364 | 1:500 |
| PSD95 | DLG4 |  | Y |  | Y | Cell Signaling | 3450 | 1:1000 |
| pSRC(Tyr416) | FYN |  | Y |  | Y | Cell Signaling | 6943 | 1:500 |
| RAPTOR | RPTOR |  |  |  |  | Cell Signaling | 2280 | 1:750 |
| RCAN1 | RCAN1 |  |  |  |  | Sigma-Aldrich | D6694 | 1:2000 |
| RRP1 | RRP1 |  |  |  |  | Aviva Systems Biology | ARP45812_P050 | 1:2000 |
| RSK2 | RPS6KA3 | Y |  |  |  | Cell Signaling | 9340 | 1:300 |
| S6 | RPS6 |  |  |  |  | Cell Signaling | 2217 | 1:500 |
| SHH | SHH | Y |  |  |  | Cell Signaling | 2207 | 1:500 |
| SOD1 | SOD1 |  |  |  |  | Santa Cruz Biotechnology | SC-11407 | 1:500 |
| SYP | SYP | Y |  |  |  | Epitomics | 1870-1 | 1:5000 |
| Tau | MAPT |  | Y |  | Y | Cell Signaling | 4019 | 1:500 |
| TH | TH | Y | Y |  |  | Cell Signaling | 2792 | 1:750 |
| TIAM1 | TIAM1 |  |  |  |  | Abcam | Ab54458 | 1:1500 |
| TRKA | NTRK1 | Y | Y |  |  | Epitomics | 2244-1 | 1:2000 |
| Ubiquitin | UBC |  |  |  |  | Cell Signaling | 3933 | 1:1000 |
| A−Synuclein | SNCA |  |  |  | Y | Cell Signaling | 4179 | 1:3000 |
| β-Catenin | CTNNB1 | Y |  |  |  | Cell Signaling | 9562 | 1:1000 |
