# Supplementary material for: Protein Dynamics Associated with Failed and Rescued Learning in the Ts65Dn Mouse Model of Down Syndrome
Source: PLoS One. 2015 Mar 20;10(3):e0119491. doi: 10.1371/journal.pone.0119491 (PMC4368539; doi:10.1371/journal.pone.0119491)
Supplement: S1 Text — (DOCX) [file pone.0119491.s004.docx]

Supplementary text. Additional protein patterns: stable proteins and proteins responding in a single comparison.

Of the proteins with detectable expression in hippocampus or cortex, 10%-15% showed no response in any of the five comparisons. These stable proteins are specific to brain region and fraction and do not reflect any specific pathway or process. For example, in hippocampus, levels of pELK are stable in both nuclear and cytosolic fractions, and levels of Hsa21 proteins DYRK1A and TIAM1 are stable in the membrane fraction. In cortex, levels of CFOS and pCREB are stable in the nuclear fraction and ITSN1, in the cytosolic. Given that measurements were made at a single time point, however, any of these proteins many have responded at different times post training.

A number of proteins in both hippocampus and cortex responded in only one comparison. In hippocampus, these 38 uniquely responsive instances were most common in NL (15 of 38), where four, eight and three changed in the nuclear, cytosolic and membrane fractions, respectively. In the cytosol, seven of eight were decreases, with the sole increase seen in pCAMKII. In B, six, four and one proteins were altered in nuclear, cytosolic and membrane fractions, and of these, four, three and one were Hsa21 encoded proteins, and others were components of the MAPK and MTOR pathways. These trisomy-associated abnormalities themselves therefore do not impair successful learning, showing that elevated levels of at least some Hsa21 gene products do not need to be corrected for successful learning. It is interesting that there are few responses unique to FL, only BAD, BCL2, CFOS and RAPTOR in the hippocampus nuclear fraction and none in cortex, i.e. potentially deleterious responses are fewer than failed responses. Unique responses were not similar in cortex: of a total of 16, nine occurred in RL and only three in NL. Overall, it must be assumed that these unique responses are either irrelevant to successful learning or that they are compensated by different molecular events in RL, or that the timing of responses in these proteins differs between controls and trisomic mice.

An additional pattern involves instances where changes occurred in both FL and RL, but in no other instances. In the nuclear fraction of cortex, of seven such instances, three were decreases in components of the MTOR pathway (MTOR, RAPTOR and AKT) and four were decreases in components of MAPK (pBRAF, pMEK, pCAMKII and CREB). No such patterns were observed in hippocampus. Given the known importance of these pathways to L/M, the lack of the same responses in NL may be an indication of altered timing of responses in trisomy vs controls or compensation for perturbations not measured here that are specific to trisomy. Another pair of responses are instances where changes in RL were equal in magnitude but opposite in direction to those in B-tm. There were four instances in hippocampus and ten in cortex. One interpretation of these is that memantine induces a change which subsequently is corrected in RL.

There remain a number of instances where biological interpretation is difficult. Examples in the nuclear fraction of hippocampus include increases in ERK of 21% and 17% in NL and B, respectively, plus a much larger increase, 70%, in RL, and decreases in pCREB of 13% and 14% in FL and RL, plus an increase in B-tm of 15%. In the cytosolic fraction of hippocampus, levels of BDNF increased by 12% and 29% in NL and FL, respectively, but remained stable in RL, and levels of NR2A increased by 32% in FL, decreased by 26% in B-tm and increased by 86% in RL.
